# The impact of thigh and shank marker quantity on lower extremity kinematics using a constrained model

**DOI:** 10.1186/s12891-018-2329-7

**Published:** 2018-11-13

**Authors:** Annelise A. Slater, Todd J. Hullfish, Josh R. Baxter

**Affiliations:** Human Motion Laboratory, Department of Orthopaedic Surgery, University of Pennsylvania, 3737 Market Street, Suite 702, Philadelphia, PA 19104 USA

**Keywords:** Motion capture, Musculoskeletal model, Constrained-kinematic model, Lower extremity

## Abstract

**Background:**

Musculoskeletal models are commonly used to quantify joint motions and loads during human motion. Constraining joint kinematics simplifies these models but the implications of the placement and quantity of markers used during data acquisition remains unclear. The purpose of this study was to establish the effects of marker placement and quantity on lower extremity kinematics calculated using a constrained-kinematic model. We hypothesized that a constrained-kinematic model would produce lower-extremity kinematics errors that correlated with the number of tracking markers removed from the thigh and shank.

**Methods:**

Healthy-young adults (*N* = 10) walked on a treadmill at slow, moderate, and fast speeds while skin-mounted markers were tracked using motion capture. Lower extremity kinematics were calculated for 256 combinations of leg and shank markers to establish the implications of marker placement and quantity on joint kinematics. Marker combinations that yielded differences greater than 5 degrees were tested with paired t-tests and the relationship between number of markers and kinematic errors were modeled with polynomials to determine goodness of fit (R^2^).

**Results:**

Sagittal joint and hip coronal kinematics errors were smaller than documented errors caused by soft-tissue artifact, which tends to be approximately 5 degrees, when excluding thigh and shank markers. Joint angle and center kinematic errors negatively correlated with the number of markers included in the analyses (R^2^ > 0.97) and typically showed the greatest error reductions when two markers were included on the thigh or shank segments. Further, we demonstrated that a simplified marker set that included markers on the pelvis, lateral knee condyle, lateral malleolus, and shoes produced kinematics that strongly agreed with the traditional marker set that included 3 tracking markers for each segment.

**Conclusion:**

Constrained-kinematic models are resilient to marker placement and quantity, which has implications on study design and post-processing workflows.

## Background

Musculoskeletal modeling relies on accurate experimental data to calculate the motions and loads generated during human motion. Despite recent advances in motion capture technology that have improved marker tracking to sub-millimeter precision, soft-tissue artifact continues to be a major limiter of the clinical efficacy of motion capture data [1]. A recent special edition of the Journal of Biomechanics proposed new and innovative techniques to mitigate some of the effects of soft-tissue artifact [2]. While these techniques improve the overall fidelity of motion capture data, they introduce new challenges to both the collection and processing workflows [3–7]. This study takes a different approach to the problem. Instead, seeking to understand how currently implemented techniques can be streamlined to preserve kinematic accuracy while reducing the burdens placed on subjects and researchers.

Unconstrained-kinematic models – often referred to as ‘six degree-of-freedom’ – are commonly utilized to quantify joint motion using skin-based motion capture [8, 9]; however, their accuracy has been challenged by fluoroscopy and bone-pin studies [10, 11]. For example, knee valgus and internal rotation errors of 117 and 192%, respectively, have been reported despite utilizing techniques that are aimed at minimizing soft tissue artifact [12]. In addition, unconstrained joints increase the complexities of musculoskeletal models, making simulation of human motion challenging.

Constrained-kinematic models leverage well-known characteristics of joint function [13, 14] to compensate for soft-tissue artifact while minimizing the number of markers needed to quantify motion [15]. These models also make possible advanced analyses of neuromuscular function and forward dynamic simulations [16] without the need of simulating joint contact, which is impractical to implement on large data sets. Despite these inherent strengths of constrained-kinematic models, experimental considerations of marker placement and quantity have not yet been associated with kinematic fidelity; specifically, whether marker placement and quantify alter lower extremity range of motion, root mean square errors, and cross-correlations when compared to a kinematic model that utilizes four markers on each segment.

The purpose of this study was to quantify the implications of marker placement and quantity on lower extremity kinematics using a constrained-kinematic model. To do this, we tested 256 combinations of marker number and placement and characterized their effects on lower extremity kinematics and joint centers – a surrogate measure of joint kinetics [17]. We hypothesized that (1) joint kinematics calculated using constrained and unconstrained models would not differ and (2) lower extremity kinematic errors (root mean square errors) would positively correlate with the number of markers excluded from the analyses. The secondary aim of this study was to identify a ‘simplified’ marker set that provides kinematic fidelity while minimizing the number of markers needed for model definition and kinematic tracking. Additionally, we tested the effects of marker sets on three different walking speeds (slow, medium, and fast) to determine if a ‘simplified’ marker set could detect subtle changes in joint kinematics. If successful, these findings will provide support to modify existing laboratory standards regarding marker placement and quantify in order to streamline subject setup and accommodate other experimental constraints – such as wearable devices, braces, and other measurement equipment.

## Methods

### Subjects and motion capture

**Fig. 1:**
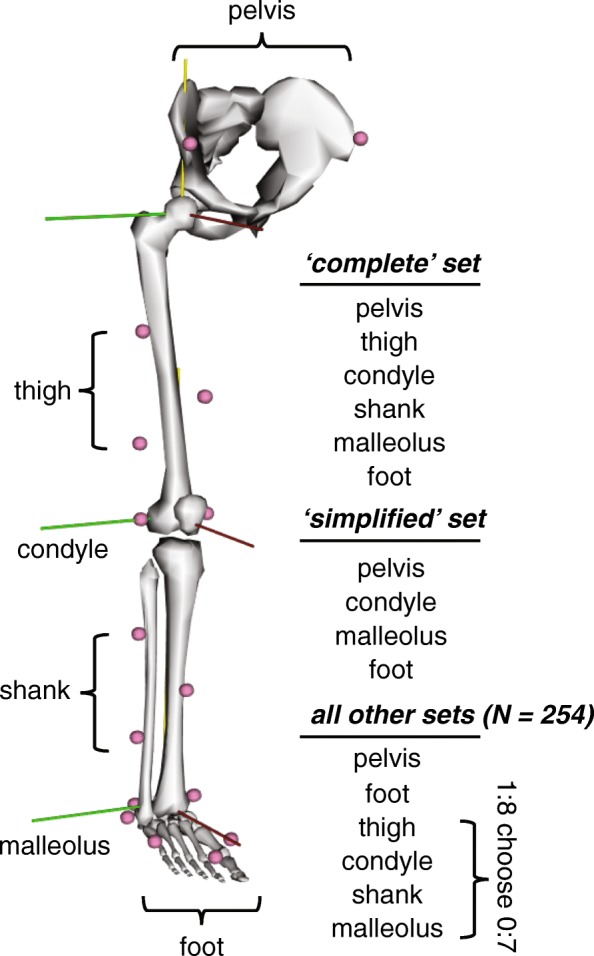
Subject-specific models (left leg hidden for clarity) were scaled based on subject anatomy and positioning. Inverse kinematics were then performed for walking trials under 256 marker combinations to test the effects of all possible marker positions and quantities attached on the thigh and shank

Motion capture was performed on 10 healthy-young adults (24 ± 4 years, 6 females, BMI 24.2 ± 3.4) who provided written consent in this IRB approved study. Subjects were excluded if they had a recent lower-extremity injury that limited their activity levels. Retro-reflective markers (9.5 mm, B&L Engineering, Santa Ana, CA) were placed on the lower-extremities of each subject and tracked using a 12-camera motion capture system (Raptor Series, Motion Analysis Corp, Santa Rosa, CA) while subjects walked on a treadmill (TMX428, Trackmaster, Newton, KS). Markers were placed over anatomic landmarks (Fig. 1) of the pelvis: anterior and posterior superior iliac spines; legs: lateral knee condyle and lateral ankle malleolus; and feet: calcaneus, first and fifth metatarsal heads, and the great toe that were placed on the shoes. Additional tracking markers were placed on the proximal-lateral (#1), distal-lateral (#2), and middle-anterior (#3) regions of the thigh and shank [18]. Marker positions were acquired while subjects stood in a neutrally-aligned position, which were used to scale a generic musculoskeletal model. Next, subjects walked on a treadmill at a slow (0.9 m/s), moderate (1.2 m/s), and fast (1.5 m/s) pace. Each trial lasted 2 min and generated approximately 100 strides for each leg. Joint angles and centers during each of the 100 measured strides were averaged over each walking speed and marker combination then compared to the kinematics calculated from the complete marker set. Heel strike events were identified using a kinematic-based algorithm [19].

### Constrained-kinematic model

Lower extremity kinematics were calculated for 256 different combinations of thigh and shank markers using a constrained-kinematic model implemented in open-source musculoskeletal modeling software (Opensim v3.3; [20]). This lower extremity model [18] – defined the hip as a ball joint, the knee as a mobile-hinge joint, the foot and ankle as an oblique universal joint, and the forefoot as a hinge joint – was scaled based on anatomic landmarks captured in the neutrally-aligned position. We used this single degree-of-freedom knee joint that proscribed non-sagittal motions [13] for two reasons: 1 – soft-tissue artifacts cause errors greater in magnitude than the actual joint motion in the coronal and transverse planes [10, 21, 22] and 2 – the muscles that cross the knee joint do have limited leverage outside of the sagittal plane. Marker trajectories were interpolated using a cubic-spline and low-pass filtered at 6 Hz [8]. Hip, knee, and ankle kinematics were calculated using inverse kinematics and all markers received equal weighting [20]. Markers on the thigh and shank segments were systematically excluded from the analysis (Fig. 1), so every combination of markers ranging from 0 to all 8 were tested (pseudocode. 1). This combinatory study tested 256 marker combinations tested to characterize the effects of marker location and inclusion on joint kinematics.

Pseudocode: for *i* = 1 to 8, for j = 1 to i-1, i choose j, endfor, endfor.

Subject-specific musculoskeletal models were scaled using a previously reported generic model [18] and marker positions captured while subjects stood in the anatomic position. The pelvis, thighs, shanks, and feet were scaled based on markers placed on anatomic landmarks: pelvis – right and left anterior superior iliac spines; thigh – anterior superior iliac spine and lateral condyle; shank – lateral condyle and lateral malleolus; and foot – lateral malleolus and toe. The scaled model was then moved to the anatomic position by fitting the model to the anatomic marker positions and recorded joint angles. The anterior superior iliac spines, lateral condyles and malleoli, heel, 1st and 5th metatarsal heads, and toe markers were all given equal weighting. Similarly, the hips, knees, ankles, and toe joints were all weighted towards neutral sagittal alignments. Since hip adduction and rotation varied amongst subjects during the anatomic pose, those coordinates received no weighting. Finally, scaled models were confirmed by superimposing the marker positions over the model.

During the pilot testing for this study (*N* = 3), we calculated the functional hip joint centers [23] and compared these locations to the hip joint centers from the scaled models [18]. We found that the functional hip joint centers were 30% wider than the generic model, which agrees with prior reports of pelvic morphology [24]. Therefore, we increased the hip joint center width in the unscaled generic model and scaled this modified model for all research subjects based on pelvis anatomy. This had appreciable effects on the initialization of models during pilot testing, where the model positioning agreed more strongly with the marker positions when the wider hip joint center locations were implemented.

### Unconstrained-kinematic model

Unconstrained joint kinematics were calculated to confirm if the unconstrained and constrained calculations yielded similar results. Anatomic coordinate systems were assigned to each segment using established definitions [25, 26] that mirrored the coordinate systems defined in the constrained-kinematic model (Fig. 1). Briefly, flexion axes were assigned to the proximal segment, internal rotation axes were assigned to the distal segment, and the shared axes of the two segments represented joint adduction. Four markers on each segment, which three ‘tracking’ markers and a distal-lateral joint marker, were used to track and define joint motions with a least squares approach to minimize the effects of soft-tissue artifact [27]. Euler rotations were calculated using a flexion-adduction-rotation sequence [26], and joint angles during the anatomic pose trial were matched with the joint angles calculated in the constrained-kinematic model in order to perform a true one-to-one comparison.

### Accounting for uncertainty associated with soft-tissue artifact

Soft-tissue artifact is an inherent limitation of marker-based motion capture. Biplane fluoroscopy studies, which are considered to be a gold standard for quantifying skeletal kinematics, have demonstrated that lower extremity kinematics quantified using motion capture vary approximately 5 degrees from true skeletal motion [1, 21]. In order to establish an equivalence between different kinematic models and marker sets, we analyzed each condition in order to detect differences in peak joint rotations and range of motions that exceeded than this 5 degrees threshold of uncertainty. In order to approximate the soft-tissue artifact in the current study, we calculated the root mean square between the experimentally collected marker trajectories and the constrained-model marker trajectories that were output from the inverse kinematics algorithm.

### Statistical analysis

Joint kinematics were first post-processed to calculate summary statistics and kinematic error data for further statistical analysis. Two primary analyses were performed: 1 – joint angles calculated using the unconstrained and constrained models that included all tracking markers and 2 – joint angles and centers for each marker combination using the constrained-kinematic model were compared to the constrained model that included all tracking markers. Joint center displacements in the anterior-posterior, superior-inferior, and medial-lateral directions were calculated with respect to joint center positions from the complete marker set. Maximal and minimal joint rotations as well as joint range of motion were calculated for hip flexion and adduction as well as knee flexion and ankle plantarflexion. Cross-correlation coefficients [28] and root mean square (RMS) errors were calculated for joint kinematics. Ninety-five percent bootstrap confidence intervals were calculated (bootci, MATLAB, The Mathworks, Natick, MA, USA) using a using the average kinematic curves from each subject [28] to demonstrate the amount of certainty in the joint kinematics and visualized in plots. Prior to data analysis, we defined a ‘substantially different’ cross correlation coefficient (*r*_*xy*_) to be less than 0.9. Hip internal rotations were also calculated as part of a secondary analysis.

To test our first hypothesis that joint kinematics calculated using constrained and unconstrained models would not differ, we determined if these kinematic models produced kinematic curves that did not differ past the 5 degree threshold. To test our second hypothesis that lower extremity kinematic errors would be positively correlated with the number of markers excluded from the analyses, we calculated the root mean square error of the kinematic curves with respect to the full marker constrained model. We then fit polynomials to these root mean square error data as a function of the number of markers included in the analysis. Additionally, we tested each marker set for differences in lower extremity kinematics between different marker combinations using the constrained kinematic model that exceeded the 5 degree threshold. Paired t-tests were performed on instances in which this 5 degree thresholds were exceeded to test for statistically significant differences (*p* < 0.05) between the full and modified marker sets. These bootstrapped confidence intervals calculated from the complete marker set data were expanded by 5 degrees to demonstrate the uncertainty associated with skin mounted markers compared to more direct techniques [1, 21]. Marker sets that produced joint kinematics that fell within the 5 degree threshold and were strongly correlated (*r*_*xy*_ > 0.90) compared to the full marker set were considered to be ‘high fidelity’.

Joint kinematics calculated at three walking speeds were compared to determine if a ‘simplified’ marker set – consisting of markers on the pelvis, lateral condyles, lateral malleoli, and shoes – detects speed-dependent changes in joint excursions similarly to a traditional marker set. This simplified marker set was selected because it is easily implemented and the markers placed on the lateral knee and ankle joints are needed to initialize the musculoskeletal model. Group means were compared using paired t-tests and corrections of multiple comparisons were not performed to decrease the likelihood of type II errors, thus making these analyses less conservative and more likely to reject the null hypothesis (no difference between marker sets) when a difference exists.

## Results

### Constrained and unconstrained kinematic models

**Fig. 2:**
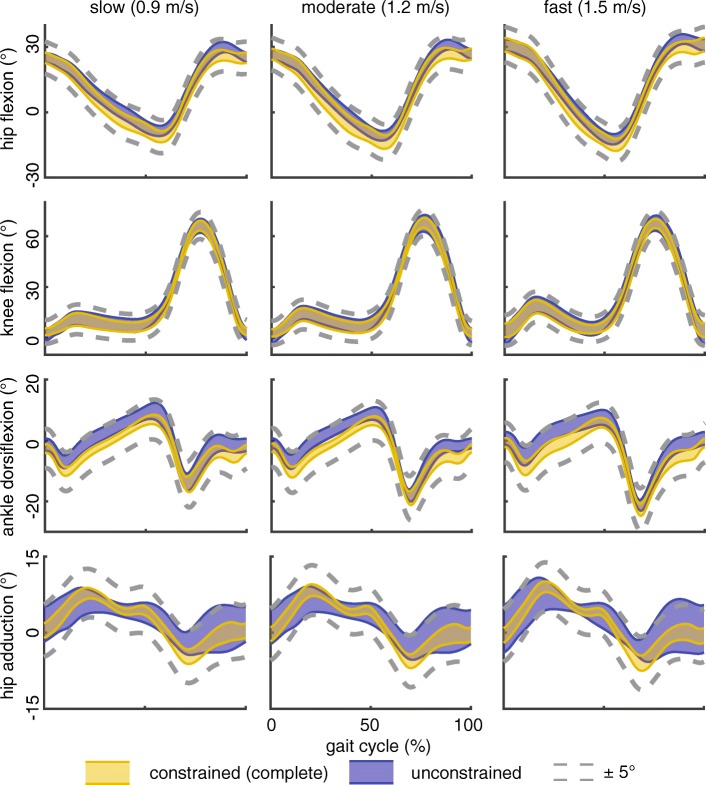
Lower extremity kinematics calculated using the constrained (gold band) and unconstrained (purple band) models were similar to within 5°. Sagittal knee and hip kinematics had the strongest agreement (*r*_xy_ ≥ 0.90), ankle sagittal kinematics fell just below our threshold for ‘substantially different’ (0.85 < *r*_xy_ < 0.90), and hip abduction was only moderately correlated between the two kinematic models (0.65 < *r*_xy_ < 0.71)

Constrained and unconstrained-kinematic models calculated sagittal plane and hip adduction kinematics that differed less than the a priori 5 degree threshold (RMS errors: 1.6–3.2°; Fig. 2). Hip and knee flexion patterns were strongly correlated (*r*_xy_ ≥ 0.90), ankle sagittal motions fell just below the cutoff value for ‘substantially different’ (0.85 < *r*_xy_ < 0.90). Hip adduction patterns were moderately correlated (0.65 < *r*_xy_ < 0.71). Despite any detected differences in kinematic patterns, joint excursions varied by less than five degrees between unconstrained and constrained models. Estimated soft-tissue artifact of markers on the thigh segment were almost twice as large as markers on the shank segment (RMS error 12.6 and 6.7 mm, respectively, Table 1).

**Table 1:** Calculated root mean square errors (mm) between experimentally and model marker trajectories using the full markers set

	Slow (0.9 m/s)	Moderate (1.2 m/s)	Fast (1.5 m/s)
Thigh1	13.0	13.5	14.2
Thigh2	9.0	10.4	10.4
Thigh3	12.3	11.8	13.7
Lateral Knee	13.0	13.6	15.7
Shank1	9.5	9.6	9.2
Shank2	5.4	5.5	5.7
Shank3	6.1	6.7	7.1
Lateral Ankle	5.0	5.2	5.7

### Effects of marker placement and quantity on kinematics

**Fig. 3:**
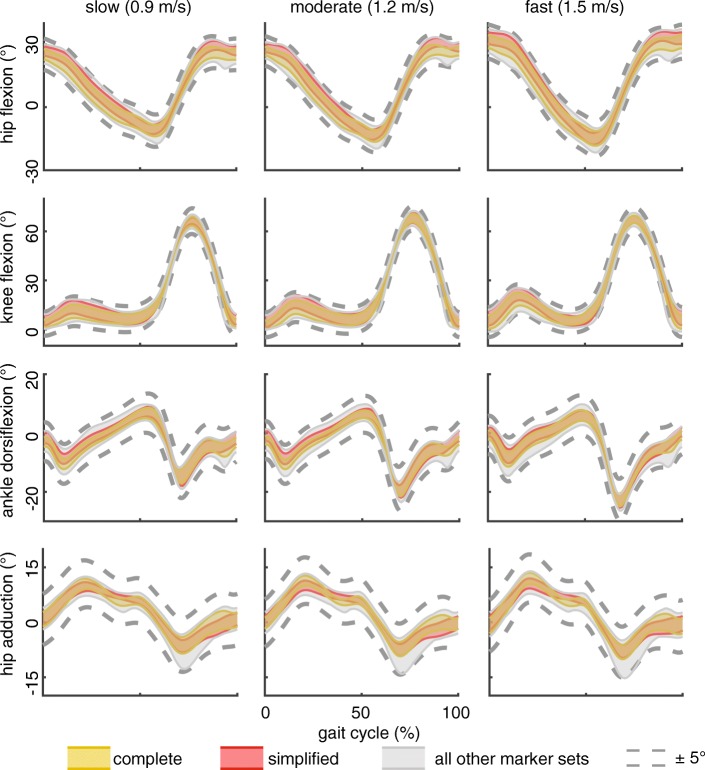
Lower extremity kinematics strongly agreed (*r*_xy_ ≥ 0.90) between the complete marker set (gold band) and all 255 other marker combinations (gray band). The 5° uncertainty threshold (dashed lines) was not exceeded by any marker combination – including the ‘simplified’ marker set (red band) – in the sagittal plane and only 2 marker sets resulted in differences in hip adduction excursion greater than 5°. 95% confidence intervals were calculated using a bootstrapping technique to characterize the variability within our study cohort (*N* = 10)

Lower extremity sagittal kinematics, calculated using the constrained kinematic model, were not strongly affected by removing thigh and shank markers from the kinematic analysis (Fig. 3). Specifically, including markers on the lateral knee condyles and malleoli generated high-fidelity sagittal kinematics compared to the constrained-kinematic model that utilized all tracking markers (*r*_xy_ ≥ 0.94; RMS errors < 2.3°). Regardless of the number of markers included in the kinematic analyses, adduction patterns were similar (0.85 < *r*_xy_ < 0.90) and joint range of motion as well as flexion and extension peaks did not deviate beyond the 5° uncertainty threshold. Hip adduction was accurately measured by all but two marker sets – when all markers proximal to the lateral malleoli were removed.

**Fig. 4:**
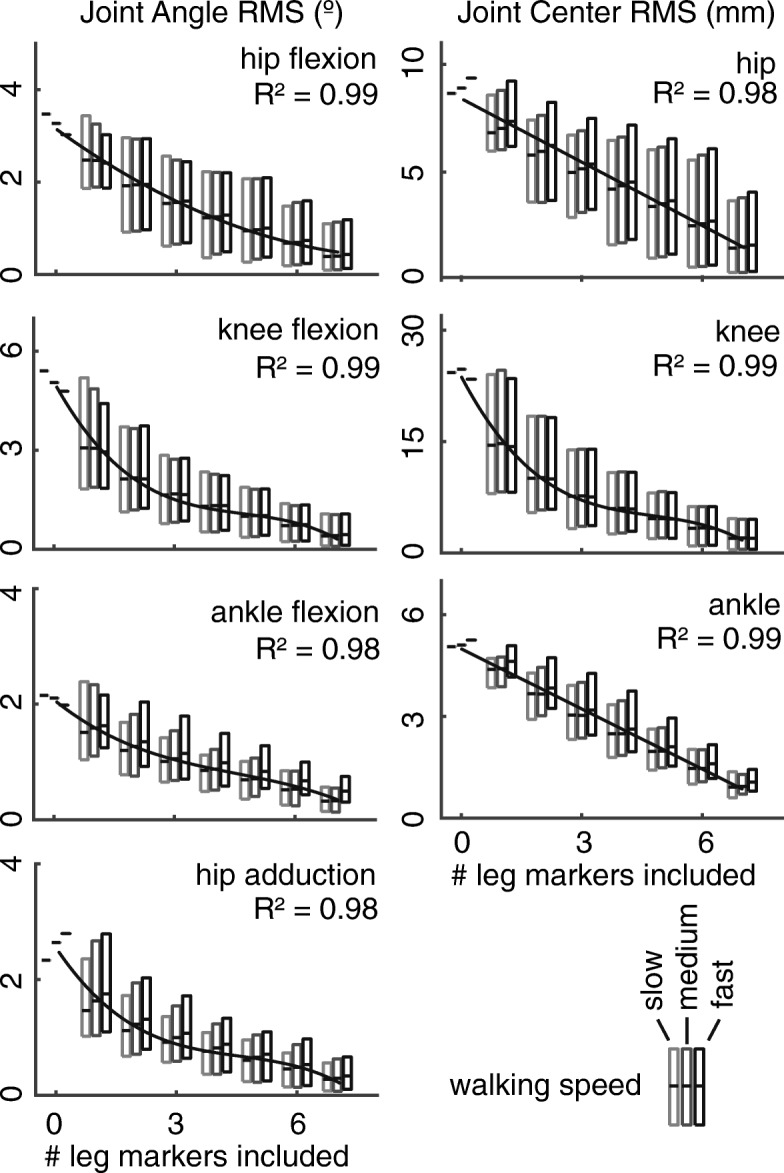
Lower extremity RMS errors negatively correlated with the number of leg markers included in the kinematic analyses. Joint kinematics (angles; left column) errors were best described by a 3rd order polynomial (R^2^ > 0.98). Knee center errors were also best characterized by a 3rd polynomial (R^2^ = 0.99; right column), while hip and ankle center errors linearly correlated with the number of leg markers included in the analyses (R^2^ > 0.98; right column). Boxes show the range of all RMS values and the dark bar indicates the average RMS value. Walking speed did not affect kinematic errors

Joint angle and center kinematic errors were negatively correlated with the number of markers included in the constrained-kinematic analysis (Fig. 4). Joint angle errors decayed at rates that were best fit by non-linear polynomials (R^2^ > 0.97, Fig. 4), where most of the errors were reduced by including two markers placed on either the thigh or shank in the kinematic analyses. Knee joint center errors were 2–4 fold greater than hip and ankle joint center errors, respectively. Including additional markers in the kinematic analyses had a strong-linear effect (R^2^ > 0.97, Fig. 4) on hip and ankle joint center errors, while knee joint center errors decayed at a cubic rate (R^2^ = 0.99, Fig. 3b) with diminishing improvements after two markers were included. Hip and ankle joint center positions were less affected by reduced markers (RMS error < 6 mm) than the knee joint (RMS error < 19 mm).

Increased joint excursions with walking speed were identified with both the complete and simplified marker sets (Table 2; Fig. 2). The complete and simplified marker sets demonstrated similar sensitivities to detecting increases in sagittal joint excursion. Similarly, hip adduction increased with walking speed; however, subtle increases of less than 2° between moderate and fast walking speeds were only detected with the complete marker set.

**Table 2:** 95% confidence intervals for lower extremity ranges of motion – calculated using the constrained-kinematic model with full and simplified marker sets – during walking at increasing speeds

	slow (0.9 m/s)	moderate (1.2 m/s)	fast (1.5 m/s)
hip flexion	(37.2–40.4) | (38.6–43.1)	(41.2–44.2)^s^ | (42.8–47.0)^s^	(44.7–49.7)^sm^ | (46.7–53.2)^sm^
knee flexion	(58.7–67.5) | (56.8–66.2)	(61.3–69.8)^s^ | (59.4–68.4)^s^	(61.7–68.5) | (60.7–67.4)^s^
ankle dorsiflexion	(20.3–24.5) | (20.9–25.5)	(23.3–28.8)^s^ | (24.2–29.5)^s^	(26.3–32.0)^sm^ | (26.7–32.5)^sm^
hip adduction	(15.2–18.7) | (14.6–18.2)	(16.5–21.0)^s^ | (15.6–20.0)^s^	(18.0–22.7)^sm^ | (16.3–21.2)^s^
hip rotation	(7.9–12.0) | (11.1–16.0)	(10.5–14.1)^s^ | (13.5–18.7)^s^	(11.4–16.2)^sm^ | (14.9–20.1)^s^

95% confidence intervals for joint range of motion for the full and simplified marker sets are reported for each joint coordinate and walking speed (full | simplified). ^s^increased range of motion compared to slow speed. ^m^ increased range of motion compared to medium speed. *p* < 0.05

Hip internal rotation patterns were weakly correlated (0.10 < r_xy_ < 0.21) with calculations using an unconstrained-kinematic model and demonstrated differences that exceed five degrees (RMS errors: 3.9–5.4°). The effects of removing thigh and shank markers from the constrained-kinematic model had moderate effects (0.55 < *r*_xy_ < 0.90). However, hip internal rotation excursions were within five degrees of the complete marker set in 95% of the marker combinations.

## Discussion

We demonstrated that constrained-kinematic models accurately reproduce lower extremity kinematics of walking as well as a complete marker set when numerous markers are excluded from the analyses. The effects of reducing markers on sagittal kinematics and hip adduction are smaller than kinematic uncertainty caused by soft tissue artifact [1, 21, 22]. Joint center trajectories, which govern the joint moment arm of the ground reaction force – and thus joint kinetics (Myers, 2015) – appear to also be resilient to decreased markers. Since marker placement minimally affects joint kinematics, researchers can tailor marker sets based on experimental constraints. For example, a ‘simplified’ marker set (Fig. 1), that excludes the traditional tracking markers adhered to the thigh and shank, can be utilized without compromising kinematic fidelity to increase motion capture workflow and provide more flexibility for the placement of other sensors and wearable devices.

Lower extremity kinematics quantified in this study compared favorably with prior reports. Similar to prior work [29, 30], we found that sagittal hip, knee, and ankle excursion increased with walking speed (Table 2). Hip coronal kinematics measured in this study demonstrated stereotypical patterns that are well described in the literature [31, 32]. Since much of the literature implements six degree-of-freedom marker sets, we calculated the unconstrained motion of the lower extremity and implemented a least squares approach [27] to minimize the effects of soft tissue artifact on resulting joint kinematics. Sagittal joint and hip coronal motions were similar between the unconstrained and constrained-kinematic results (Fig. 2). Hip internal rotation differed between the unconstrained and constrained model, which may be explained by well documented soft tissue artifact of the thigh segment [33]. However, these differences were less pronounced between constrained marker sets, likely due to the lack of knee rotation in the musculoskeletal model.

**Fig. 5:**
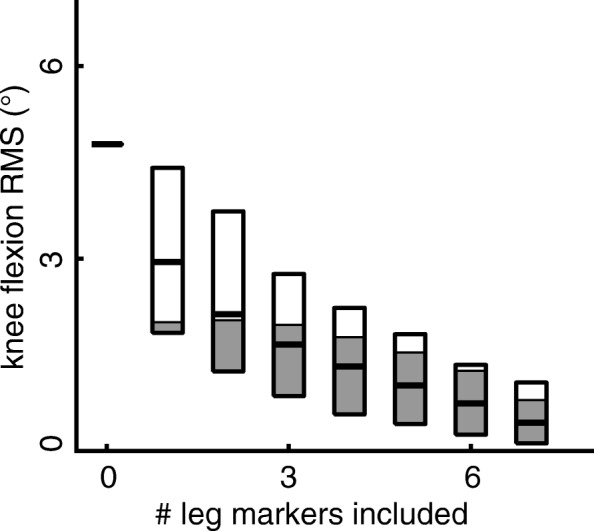
Constrained-kinematic models that included lateral knee condyle marker (shaded part of box) effectively decreased the kinematic errors compared to marker sets that excluded the knee marker (unshaded part of box) when compared to the full marker set. Boxes show the range of all RMS values and the dark bar indicates the average RMS value

Our findings demonstrate that constrained-kinematic models are resilient to marker placement and dropout. Although we did not directly track skeletal motion in this study, we approximated the uncertainty introduced by soft-tissue artifact by calculating the difference between experimentally-measured marker and model-fixed marker trajectories. We found that the markers placed on the thigh, lateral knee, and shank had average RMS values of 12.5, 14.1, and 7.1 mm, respectively (Table 1); compared to direct measurements of soft-tissue artifact in the literature of 13.8, 13.9, and 10.8 mm, respectively [21]. Despite the lateral knee being prone to soft-tissue artifact, its inclusion improved kinematic tracking when fewer than five leg markers were included in the kinematic analyses (Fig. 5). Increasing the number of markers used for gait analysis has diminishing returns with regard to lower extremity kinematics (Fig. 4).

Excluding all of the markers attached to the thigh and shank generated sagittal joint kinematics that were in strong agreement with the complete marker set but adversely affected knee joint center kinematics, which impacts joint loads [17]. Adding markers to the lateral condyles and malleoli – which were used to scale the musculoskeletal model – mitigated the majority of kinematic errors (*r*_xy_ ≥ 0.94; RMS errors < 2.3°). To improve experimental consistency and workflow, markers can be permanently fixed to lab shoes, which reduces the number of markers applied to the subject to eight: four on the pelvis and two on each leg. Thus, a ‘simplified’ marker set accurately characterizes joint kinematics and joint center motions by providing essential inputs to constrained-kinematic models.

Changing the placement and quantity of tracking markers can reduce experimental setup time, allows for more comfortable attire to be worn during data collection, and provides fewer obstructions for other experimental equipment while being resistant to errors kinematic errors (Figs. 3, 4 and 5). While unconstrained-kinematic models require at least three markers on each segment at all times, our results demonstrate that constrained-kinematic models can perform well with no markers on certain segments; for example, the thigh and shank. Hierarchical marker sets track segment kinematics by assuming the location of a joint center based on a nearby segment [34]. However, this approach is susceptible to soft-tissue artifact [35] and does not provide the necessary joint constraints for advanced musculoskeletal analyses [36]. Our findings also benefit researchers utilizing wearable-assistive devices [37, 38], ultrasonography during human motion [39], and high-density electromyography sensors [40] – all techniques that require unobstructed access to the lower extremities.

Processing and analyzing motion capture data can be streamlined into a turn-key routine utilizing open-source musculoskeletal modeling software [20] and batched scripts. In addition to calculating joint kinematics, constrained-kinematic models are well-suited for performing both inverse and forward dynamic simulations. Integrating gait analysis into a single musculoskeletal modeling environment provides investigators with a standardized workflow while maintaining the flexibility needed to perform specific analyses [41, 42]. Further, many analyses are not possible to perform without imposing joint constraints or contact [43, 44]. Therefore, migrating kinematic analyses into a constrained-kinematic model may minimize workflow complexity without compromising kinematic fidelity (Fig. 2).

Several limitations should be considered when interpreting these findings. We did not directly measure skeletal motion but did show similarities in joint kinematics with prior studies that utilized intracortical bone pins and fluoroscopy [21, 45]. Instead, we demonstrated that both the constrained and unconstrained kinematic models produced equivalent lower extremity kinematics, based on the fact that these kinematics were within a previously determined 5 degree threshold [1, 21]. Subject walked at three different speeds but did not perform more dynamic tasks such as jump-cut that are associated with large errors due to soft-tissue artifact [46], which limits the study findings to lower impact activities like walking. Subjects in the present study were healthy-young adults that were generally fit with a healthy body mass index (BMI 24.2 ± 3.4), which may not be representative of clinical populations. Joint kinematics are sensitive to joint-axis location and orientation [47, 48], which may be affected when scaling generic musculoskeletal models to subject-specific anthropometry. To mitigate these potential errors, we visually confirmed that each subject-specific model closely matched the neutrally-aligned position. Further, we confirmed joint kinematics using unconstrained-kinematic models that shared the same joint axis definitions as the constrained-kinematic models (Fig. 2). Due to knee valgus and internal rotation errors as high as twice that of skeletal motion [12], we limited knee joint kinematics to a single degree-of-freedom and prescribed other rotations and translations based on flexion angle [13]. Accurately measuring frontal plane knee kinematics during gait requires advanced imaging or invasive techniques [21], which was outside of the scope of this study. Walking trials were acquired on a commercial treadmill that did not have an integrated force plate, so we were unable to calculate joint reaction moments. We instead decided to quantify the changes in the joint center trajectories, which governs the ground reaction force moment arm and thus joint moments. In order to show the robustness of the constrained-kinematic model, we chose not to modify the hip joint center locations based on subject-specific functional hip joint locations. However, hip kinetics are sensitive to joint center location and employing more rigorous scaling techniques should be considered when high-fidelity hip kinetics are required.

## Conclusion

Constrained-kinematic models provide the flexibility to change the position and quantity of tracking markers used during gait analysis. Experiments can be designed to attain the lower-extremity kinematic fidelity necessary to answer specific research questions while adjusting marker placement and quantity to suit the constraints of the experimental setup. In addition, integrating constrained-kinematic models into a gait analysis workflow offers several advantages that can improve post-processing efficiency while providing access to unique analysis tools to test specific questions. However, investigators should weigh the strengths and weaknesses of both constrained and unconstrained-kinematic models to determine which approach is best suited for the specific research question.

## Abbreviations

BMI: body mass index
RMS: root mean square
*r*_*xy*_: cross correlation coefficient
